# Neurofilament Light and Cognition in community dwelling non‐Hispanic Blacks

**DOI:** 10.1002/alz.089504

**Published:** 2025-01-09

**Authors:** Lubnaa R Abdullah

**Affiliations:** ^1^ University of North Texas Health Science Center, Fort Worth, TX USA

## Abstract

**Background:**

No large‐scale characterizations of neurofilament light chain (NfL) and cognitive outcomes have been conducted in community dwelling non‐Hispanic Blacks.

**Method:**

Baseline data were analyzed among n = 283 non‐Hispanic Blacks (NHB) from the multi‐ethnic Health and Aging Brain Study‐ Health Disparities (HABS‐HD). Plasma NfL was measured on the Simoa platform. Linear regression models were conducted, covarying for age, gender, and education.

**Result:**

The majority of study participants (72%) were Cognitively Unimpaired (CU), with 21 % having mild cognitive impairment (MCI), and 6.7 % with Alzheimer’s Disease (AD). In adjusted models among the entire sample, significant associations existed between NfL and Trails A (P < .0001), Trails B (p < .0001), phonemic (FAS) and semantic (Animals) fluency (p = 0.03), and Symbol Digit Substitution (p < .001). When separated by diagnostic classification, significant associations were removed for functions involving executive functions for all diagnostic groups. Higher levels of NfL were positively associated with cognitive diagnosis, older age, and less education.

**Conclusion:**

Plasma NfL levels are significantly associated with measures of executive functioning, which elucidate NfL as a non‐specific marker of neurodegeneration associated with efficiency of brain functions involving attention, processing, and generativity. NfL may be a sensitive measure for the detection of alterations in cognitive processing before the onset of phenotypic functional changes of neurodegeneration.

